# RCN4GSC Workshop Report: Modeling a Testbed for Managing Data at the Interface of Biodiversity and (Meta)Genomics, April 2011

**DOI:** 10.4056/sigs.3146509

**Published:** 2012-09-24

**Authors:** Robert J. Robbins, Guy Cochrane, Neil Davies, Peter Dawyndt, Renzo Kottmann, Leonard (Kris) Krishtalka, Norman Morrison, Éamonn Ó Tuama, Inigo San Gil, John Wooley

**Affiliations:** 1 University of California San Diego, La Jolla, California, USA; 2 European Molecular Biology Laboratory (EMBL) Outstation, European Bioinformatics Institute (EBI), Wellcome Trust Genome Campus, Hinxton, Cambridge, United Kingdom; 3 Richard B. Gump South Pacific Research Station, University of California Berkeley, PO Box 244, 98728 Moorea, French Polynesia; 4 Department of Applied Mathematics and Computer Science, University of Ghent, Ghent, Belgium; 5 Microbial Genomics Group, Max Planck Institute for Marine Microbiology & Jacobs University Bremen, Bremen, Germany; 6 University of Kansas Natural History Museum, Lawrence, KS, USA; 7 School of Computer Science, Kilburn Building, University of Manchester, Oxford Road, Manchester, UK M13 9PL; 8 Global Biodiversity Information Facility, GBIF Secretariat, Copenhagen, Denmark; 9 LTER Network Office, Department of Biology, University of New Mexico. Albuquerque, New Mexico, USA

## Abstract

At the GSC11 meeting (4-6 April 2011, Hinxton, England, the GSC’s genomic biodiversity working group (GBWG) developed an initial model for a data management testbed at the interface of biodiversity with genomics and metagenomics. With representatives of the Global Biodiversity Information Facility (GBIF) participating, it was agreed that the most useful course of action would be for GBIF to collaborate with the GSC in its ongoing GBWG workshops to achieve common goals around interoperability/data integration across (meta)-genomic and species level data. It was determined that a quick comparison should be made of the contents of the Darwin Core (DwC) and the GSC data checklists, with a goal of determining their degree of overlap and compatibility. An ad-hoc task group lead by Renzo Kottman and Peter Dawyndt undertook an initial comparison between the Darwin Core (DwC) standard used by the Global Biodiversity Information Facility (GBIF) and the MIxS checklists put forward by the Genomic Standards Consortium (GSC). A term-by-term comparison showed that DwC and GSC concepts complement each other far more than they compete with each other. Because the preliminary analysis done at this meeting was based on expertise with GSC standards, but not with DwC standards, the group recommended that a joint meeting of DwC and GSC experts be convened as soon as possible to continue this joint assessment and to propose additional work going forward.

## Background

In March of 2011, a planning meeting on managing data at the interface between biodiversity and (meta)genomics was held at the University of California at San Diego [1]. The recommendations of that planning meeting were brought to the GSC11 meeting, held 4-6 April 2011 at the Wellcome Trust Conference Centre in Hinxton England.

The recommendations were presented in plenary session of the full GSC meeting, then discussed at length in breakout sessions of the GSC Genomic Biodiversity Working Group (GBWG). This report summarizes the discussion, analysis, conclusions, and recommendations that occurred on this topic at the GSC11 meeting, particularly in the GBWG breakout sessions.

Also, in early 2011, the Global Biodiversity Information Facility (GBIF) had independently issued a “Request for proposals to draft a GBIF position paper on the publishing and discovery of, and access to, primary biodiversity data in the form of genomic level observations” [2] with a submission date in March; this request was later withdrawn. Given our knowledge of their interests, GBIF was subsequently invited to attend the GSC11 meeting, so that mutual interests could be explored.

## Purposes of the Meeting

The purposes of the meeting were to present the GBWG planning-meeting recommendations to the full GSC plenary session for input, and then to hold GBWG breakout sessions to consider the recommendations in depth and form task groups as necessary.

## Participants

Participants included members of the GBWG and other participants at GSC11, including attendees from GBIF, who are especially interested in this activity.

## Activities and Analysis

The recommendations of the planning-meeting were considered during the first GBWG breakout session. The GBIF call for a white paper on genomic level biodiversity observational data was again discussed and it was agreed that the most useful course of action would be for GBIF to collaborate with the GSC in its ongoing GBWG workshops to achieve common goals around interoperability/data integration across (meta)-genomic and species level data. It was determined that a quick comparison should be made of the contents of the Darwin Core (DwC) and the GSC data checklists, with a goal of determining their degree of overlap and compatibility.

During first breakout, GBWG members introduced the history and mission of this working group to sixteen participants who represented other GSC communities; namely, biodiversity data managers (GBIF), genomic data centers (such as Genbank), (meta)genomics researchers (UCSD, Moorea, MPI, etc), commercial representatives and museums and collections (Smithsonian, Estonia). The working group chair presented the developments and recommendations reached at the March meeting at UCSD, and set the context for the next working group session.

An ad-hoc task group lead by Renzo Kottman and Peter Dawyndt undertook an initial comparison between the Darwin Core (DwC) standard used by the Global Biodiversity Information Facility (GBIF) and the MIxS checklists put forward by the Genomic Standards Consortium (GSC) and implemented in GCDML. In this group, some of the GSC standards developers were present and one of the members had some basic familiarity with the Darwin Core standard. Thus, the analysis of DwC concepts was based on a non-expert assessment of on-line documentation and must be considered only preliminary.

**A second working group session served as a forum for discussions of topics such as**
- The differences between the observation and the event concept as interpreted by members of the biodiversity communities
- The challenges associated with versioning of the metadata records. How different institutions approach data and metadata revisions, and examples of uses in several repositories.
- Standards compliance and best practices

The second part of this second working group sessions was devoted to discuss the preliminary results of the overlap and concept coverage by the DwC and MIxS, attained at the ad-hoc session.

## Conclusions

**Figure 1:**
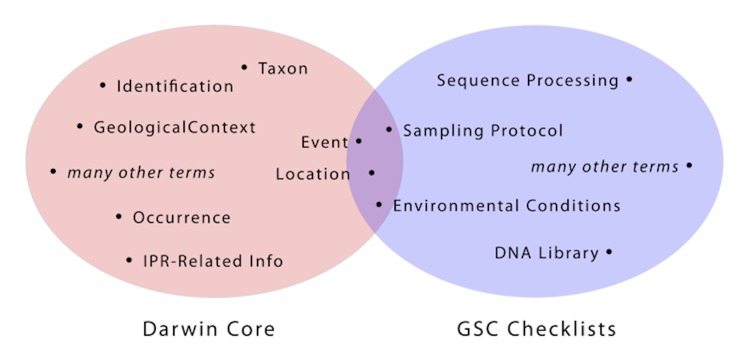
Summary comparison of the relative overlap between terms of the Darwin Core and GSC. The two sets of concepts are generally disjoint and complementary, rather than overlapping and competitive.

The first question that needed to be answered was whether DwC and GSC behave as overlapping or orthogonal (complementary) standards. A term-by-term comparison showed that DwC and GSC concepts complement each other far more than they compete with each other (Figure 1). Although this is not surprising (DwC is focused on the description of observational biodiversity data, whereas the scope of the GSC checklists is genomics and metagenomics data), it is highly desirable that a union set of terms and concepts could be created without requiring major internal revisions to either individual set.

Where both standards overlap, DwC is usually more detailed. Prime exceptions are the DwC terms *SamplingProtocol* and those within the category of *MeasurementOrFact* (which can include environmental conditions) that have been worked out into more detail in the GSC checklist. Compared to the general scopes of DwC and GSC outlined above, this might arise since the GSC standards are used for inferring insights at a molecular level and aim to make molecular data reusable for comparative studies (e.g., sampling protocol is more detailed in GSC because in metagenomic studies the sampling and processing can affect what taxa are discovered in the sample — for metagenomic datasets to be comparable, the sampling protocols for each need to be well documented), whereas the DwC standard is more generally applied and targetted at the organismal level, and then, most often for higher organisms.

It was also observed that GSC checklists seem to have a different and more intense approach to using ontologies. Compared to DwC, the MIxS uses different ontologies to specify the precise terms to be used to fill out checklist item. From this analysis, the breakout group recommended the GSC community should use DwC terms for specific GSC sections that are covered in DwC and use GCDML terms for sections not covered by DwC. In particular, this concerns the DwC categories (classes) Record-level Terms (inc IPR related information), *Occurrence, Event, Location, GeologicalContext, Taxon, Identification, Taxon*. Only *Event* and *Location* have been covered by the GSC checklists, albeit in less detail. The DwC *Taxon* sections have deliberately been left out of the GSC checklists as they were already covered in the sequence records of the International Nucleotide Sequence Database Collaboration (INSDC) and the GSC checklist was designed as an extension to the information already covered.

The conclusion that the GSC checklist elaborates more on sampling protocol and environmental conditions — details of which are extremely important in light of comparative microbial genomics — was somewhat surprising in the sense that these topics perfectly fit within the scope of DwC. The participants recommended that a discussion should be started with DwC experts and designers to see how a joint approach could be developed.

Finally, the significance of the GBWG initiative was recognized by the general attendees at the GSC11 meeting and by the GSC Board, resulting in a decision to allocate substantial time to GBWG presentations and activities (i.e., not in a parallel session) at the coming GSC12 meeting (September 2011 in Bremen, Germany).

In a more general context, we note that one of the long-standing challenges for integrating data has been that communities differ in the granularity with which they collect data - cf., the old notion that those who live in the Arctic have more words for snow than do those who live in more temperate climates - making it historically difficult to achieve political consensus for developing *de novo* data-exchange standards: any system that is *sufficiently complex* to meet the needs of *all* groups simultaneously is invariably seen as *too complex* by each individual community.

**The present observation of little overlap between DwC and GSC concepts suggests that achieving globally useful standards for data and metadata may in fact be possible, provided it occurs following this general sequence:**
- First, individual communities develop and refine data standards to meet the needs of their users.
- Then, when a need arises for data exchange between the communities, the relevant data standards are analyzed to determine their relative orthogonality (as in the Venn diagram of Figure 1).
- Finally, a joint effort is undertaken, involving data-standards experts from the two communities, to analyze formally the relationship between the standards, then to develop a union set of terms and concepts, aggregated across the disjoint components and harmonized in the intersection.
- Repeat as necessary, as more communities become engaged.

This method can succeed where *de novo* efforts have failed because it involves the development of standards (both intra- and inter-community standards) to meet immediate actual needs, rather than the commitment of scarce resources to meet possible future needs.

Harmonization must be done effectively. For example, one could harmonize the intersection only, i.e., develop guidelines for using only subsets of resolution as needed by one community, but establish the capability for others to use the full standard to expand the annotation without adversely affecting the initial annotation. Doing so would result in standards that simultaneously meet the needs of both local and global users. This approach, if applied repeatedly and systematically, would yield standards that would be capable of meeting the changing needs of the scientific communities. It would, however, require a recognition of constant change and thus to be fully effective would necessitate the development of additional approaches (e.g., internal data-standard version documentation) that could allow comparability of data collected and documented at different times, using different versions of the data standards.

Although systematically accommodating change over time adds additional complexity, it must be noted that this approach would not *create* the need for methods to support data-standard versioning — it just forces the *recognition* that such a need exists.

## Recommendations

Given that this was only a comparison, the breakout group also made some additional recommendations and outlined some future work to be done. It was clear from reading the DwC documentation that the *Event* and *Location* sections are worked out in much detail and are based on solid theoretical foundations. However, some of the details were not immediately clear from a first inspection of DwC. Therefore, it was recommended that a DwC expert should be invited during the next GSC12 meeting to give a best practice workshop on the use of the *Event* and *Location* sections from the DwC standard.

In addition, the breakout group would like to further explore the ecosystem of tools and extension mechanism built around DwC. These tools are largely unknown (and so unused) in the (meta)genomics community, but might also be applicable in that field rather than the field reinventing the wheel for their purposes. Of prime relevance here are the GBIF vocabularies server [3] and the suite of data publishing tools and supporting documentation and guides [4,5]. GBIF members with expertise in the DwC ecosystem could also be invited during GSC12 in order to bring the GSC community up to date on this matter. Another issue to look at further is the use of the *Occurrence* term in DwC and the *Environmental Ontology* (EnvO) used by the GSC checklist. It seems that for this term, there is a semantic mismatch between the two standards that needs to be sorted out. The breakout group will also finalize a term-by-term mapping between the DwC and GSC checklist and publish this information on the GSC wiki for further use. There is also scope for the two communities to cooperate on the development, maintenance and governance of shared vocabularies and ontologies.

Although it was observed that in theory the scopes of DwC and the GSC checklists are quite complementary, it remains to be seen whether there are practical case studies that can benefit from using both standards simultaneously.

The breakout group of the biodiversity session therefore proposed to set up a framed experiment that could be entitled the “Microbial Earth Catalog”. The idea is to collect meta-information about all bacterial and archaeal type strains and their complete genome sequences if available, and mold it into DwC and GCDML formats. As a rough estimate, there are about 11,000 bacterial and archaeal species with 8,000 type strains (showing some synonymy among the species). The complete genome sequence of about 800 type strains is publicly available from the INSDC databases, of which about 100 have been published in Standards in Genomic Sciences (SIGS) — the journal of the GSC.

If formally accepted, this experiment could be set up as a GSC project, undertaken as a joint initiative between the GBIF and GSC communities. Having such integrated catalog information would directly support the “Microbial Earth Project” — an established GSC project with the aim to sequence the complete genome of all bacterial type strains — and could open the door to integrate microbial information into the GBIF portal, which for the moment only has limited coverage for this domain of life. This initial outline for an integrated DwC/GCDML case study found immediate interest within the GSC community, with proposals to extend it later on to cover fungi and metagenomes. GBIF is liaising with UNITE [6] (the fungal rDNA ITS sequence database) to explore serving of its data to the GBIF network via DwC. Potential conflicts between publishing such a catalog and SiGS policy remain to be discussed within the GSC board.

Because the preliminary analysis done at this meeting was based on expertise with GSC standards, but not with DwC standards, the group recommended that a joint meeting of DwC and GSC experts be convened as soon as possible to continue this joint assessment and to propose additional work going forward.

## GBWG Timeline for 2011

Efforts by the GBWG to facilitate the development of useful data standards and procedures for the interface of biodiversity with genomics and metagenomics will be an ongoing activity. Correspondingly, we provide a timeline of events. Italics indicate that the suggested activity has already occurred; plain text indicates that the activity is proposed.

Mar: *Convene a GBWG planning meeting to initiate an analysis of biodiversity, genomics, and meta-genomics: opportunities and challenges.*
Apr: *Introduce the GBWG initiative at GSC11 meeting, UK; invite the development of use cases.*
May: Form an RCN Working Group with GSC and Darwin Core specialists
Jul: Engage with DNA barcode standard through Consortium for the Barcode of Life working group.
Sep: Report and discuss progress on initiative at GSC12 meeting, Bremen, Germany.
Oct: Engage GBIF and EOL before and during TDWG meeting, 16-21 October, in New Orleans, Louisiana, US.
Nov: Discuss metadata capture, ecological sampling and analysis, NEON workshop, Boulder, CO.
Dec: Present and discuss initiative at Fourth International Barcode of Life Conference, Adelaide, Australia.
